# Digital Dentistry: The Revolution has Begun

**DOI:** 10.2174/1874210601812010059

**Published:** 2018-01-31

**Authors:** Francesco Mangano (Guest Editor)

## Digital Dentistry: The Revolution has Begun

The digital revolution is changing the dental profession.

Intraoral, desktop and face scanners, Cone Beam Computed Tomography (CBCT), software for Computer Assisted Design Computer Assisted Manufacturing (CAD/CAM) and guided surgery, new aesthetic materials, milling machines and 3D printers are radically transforming the dental profession.

The modern digital workflow consists of four phases that are closely dependent on each other: the acquisition of information, processing that information in a project, producing the necessary devices and the clinical application on the patient. These phases integrate into the traditional workflow based on anamnesis, clinical examination, 2D radiology, treatment plan formulation, and execution of the therapies, but all based on 3D perspective, leading us to the virtual patient.

Now we can, through an intraoral scan of one or more prosthetic preparations with powerful intraoral scanners, acquire 3D information for the realization of a prosthetic Computer Assisted Design (CAD) project; within the modeling software We can design our restoration that is then milled in a highly aesthetic material (ceramic, lithium disilicate, zirconia) and applied on the patient. The same goes for implants, without having to take conventional physical impressions with impression trays, which our patients have never appreciated. At the same time, information related to teeth and gingiva, received from an intraoral scan, can be superimposed on the bone-related information acquired *via* low dosage radiation Cone Beam Computed Tomography (CBCT). It is therefore possible to plan the optimal positioning of implants with software to guide the surgery. Planning data are transferred to a surgical template that can be physically fabricated in various ways and different materials. This guide will help the surgeon correctly position the implants, without the need to raise a flap.

However, fixed prosthesis and implant surgery are not the only disciplines affected by the digital revolution; aesthetic dentistry, orthodontics and regenerative and maxillofacial surgery have also experienced this change. In aesthetic dentistry, the so-called digital smile design techniques, which can design and realize the patient's smile through 2D (digital photography) 3D (intraoral and face scanning, CAD software and milling) tools are very successful. Similarly, the application of digital techniques opens up new horizons in orthodontics, which is due to greater possibilities for diagnosis and planning (through the “safe bone” set-up), but above all, due to possibility of clinically implementing and employing a whole range of customized devices such as aligners. Regenerative bone surgery can apply personalized bone synthetic grafts on a patient's defect, because they are drawn in 3D from CBCT. The benefits for the clinician are many, such as the greater simplicity of surgery and reduction of surgical time. In the future, these bone grafts will be printed in 3D and loaded with growth factors or the patient's stem cells to accelerate and enhance healing processes. This is the perspective of Bone Tissue Engineering. Lastly, in maxillofacial surgery, it is now possible to draw and realize, by means of rapid prototyping techniques such as laser sintering and laser melting, personalized metal devices and custom-made implants, even in titanium.

All these changes represent a true revolution for our profession, comparable to what was, over 30 years ago, the introduction of dental implants.

The digital revolution opens up interesting scenarios and possibilities, but it also represents a challenge for the dentist and his/her team. In fact, it is necessary to learn about new devices, software and machines and to understand how to integrate them efficiently into the workflow.

In this special issue, entirely dedicated to the world of digital dentistry, we have gathered several scientific and clinical papers that deal with digital topics. We hope you will find them interesting and useful.

